# Gestational diabetes mellitus is associated with decreased adipose and placenta peroxisome proliferator-activator receptor γ expression in a Chinese population

**DOI:** 10.18632/oncotarget.23043

**Published:** 2017-12-08

**Authors:** Yu Gao, Ruilian She, Wenqiong Sha

**Affiliations:** ^1^ Department of Obstetrics and Gynecology, The Second Clinical Medical College of Jinan University, Shenzhen People’s Hospital, Shenzhen, China

**Keywords:** gestational diabetes mellitus, PPARΓ, adipose, placenta, glucose

## Abstract

Peroxisome proliferator-activated receptors γ (PPARγ) is a member of nuclear receptor superfamily, and studies have demonstrated that dysregulation of PPARγ was associated with gestational diabetes mellitus (GDM), which is one of the most common metabolic abnormalities occurring during pregnancy. However, the results regarding the associations between PPARγ and GDM were conflicting among different studies. The present study aimed to determine the expression of PPARγ in adipose and placenta from GDM women in a Chinese population and to further explore the role of PPARγ in GDM women. The adipose and placenta tissues were isolated from GDM women and healthy pregnant women at term. The mRNA and protein expressions of PPARγ in adipose and placenta tissues were determined by qRT-PCR and western blot, respectively. Univariate correlation analysis was used to analyze the relationship between PPARγ expression and clinical characteristics of patients. The levels of tryglycerides and HbA1c were significantly higher, while the levels of low density lipoprotein (LDL) cholesterol, adiponectin and insulin were significantly lower in the GDM women than that in the healthy pregnant women. The mRNA and protein expression of PPARγ in both adipose and placenta from GDM women were significantly lower than that from healthy pregnant women. PPARγ mRNA expression in both adipose and placenta positively correlated with LDL cholesterol and adiponectin levels, and negatively correlated with tryglycerides and glucose levels at 0 h, 1 h and 2 h of 75 g oral glucose tolerance test. In summary, our results suggest that PPARγ may be a key modulator in the development of GDM, due to the roles of PPARγ in glucose homeostasis and adipose tissue development and function.

## INTRODUCTION

Gestational diabetes mellitus (GDM) is one of the most common metabolic abnormalities occurring during pregnancy, and affects 1% to 14% of all pregnant women depending on ethnic group and the diagnostic test employed [1]. GDM is defined as glucose intolerance with onset or first recognition during pregnancy [2]. Studies have demonstrated that GDM was associated with various complications in both mother and newborn. Up to date, several factors contributed to GDM have been identified, such as altered plasma adipokine levels, inflammation, deregulation of insulin signaling pathway, oxidative stress [3-6]. Unfortunately, the precise mechanisms underlying the pathophysiology of GDM are not fully understood.

Due to the regulatory roles of peroxisome proliferator-activated receptors γ (PPARγ) in glucose and lipid metabolism, adipocyte differentiation, and inflammation, PPARγ has been shown to be associated with type 2 diabetes mellitus in a large number of studies [7-9]. Studies showed that PPARγ involved in the regulation of genes related to lipid synthesis and storage, adipokine production, and insulin signaling [10, 11]. Activation of PPAR γ by its agonist, thiazolidinediones, improved insulin sensitivity in insulin-resistant animal models and diabetic patients [12]. Mutation of human PPARγ gene has been found to be associated with increased insulin resistance, hypertension and diabetes [13]. In addition, PPARγ can also function in suppressing the production of monocyte and macrophages inflammatory cytokines such as TNF-α, IL-1β and IL-6 [14]. In the patients with GDM, the PPARγ was found to be down-regulated in both placenta tissues and adipose tissues [15, 16]. On the other hand, study investigating the mRNA expression of PPARγ in leukocyte showed that PPARγ was up-regulated in patients with GDM and positively correlated with glucose concentrations at 1 h and 2 h of 75 g oral glucose tolerance test (OGTT) and also negatively correlated with plasma high density lipoprotein (HDL) cholesterol concentration [17]. The conflicting results regarding the PPARγ expression in different tissues examined suggest the complex mechanisms of PPARγ in GDM.

Though the expression of PPARγ in adipose and placenta from patients with GDM has been demonstrated in separated studies, the association between PPARγ expression and the clinical characteristics has not been examined so far. In the present study, the expression of PPARγ was examined in both adipose tissues and placenta tissues from both healthy pregnant women and GDM women. The present study also measured the clinical characteristics in the recruited pregnant women, and we for the first time investigated the relationship between PPARγ expression in both adipose and placenta tissues and the relevant clinical parameters from a GDM women in a Chinese population. In addition, the underlying mechanisms in PPARγ-involved in GDM were discussed.

## RESULTS

### Clinical features of healthy pregnant and GDM subjects

The clinical parameters of 38 healthy pregnant women and 66 women with GDM were examined and the results were shown in Table 1. There were no significant differences between healthy pregnant women and GDM women regarding age, pre-pregnancy body mass index (BMI), pregnancy BMI, body weight gain, gestational age at delivery, fetal weight, total cholesterol, high density lipoprotein (HDL) cholesterol, apoplipoprotein A1, apoplipoprotein B, quantitative insulin sensitivity check index (QUICK-IS). The levels of tryglycerides and HbA1c were significantly higher in the GDM women than that in the healthy pregnant women; while the levels of LDL cholesterol, adiponectin and insulin were significantly lower in the GDM women than that in the healthy pregnant women. As expected, the OGTT results showed that the levels of blood glucose at 0 h, 1 h and 2 h of 75 g OGTT were significantly higher in the GDM women than that in the healthy pregnant women.

**Table 1:** Clinical parameters between healthy pregnant and GDM subjects in the present study

Parameters	Healthy group (n = 38)	GDM group (n = 66)	P value
Age (years)	31.2 ± 5.4	31.5 ± 6.5	0.8104
Pre-pregnancy BMI (kg/m2)	24.2 ± 4.5	25.6 ± 6.1	0.2202
Pregnancy BMI (kg/m2)	28.7 ± 4.9	29.3 ± 7.2	0.6493
Body weight gain (kg)	11.5 ± 4.7	9.6 ± 6.5	0.1175
GA at delivery (wks)	37.6 ± 3.4	37.4 ± 2.9	0.7513
Fetal weight (g)	3471 ± 147	3511 ± 231	0.3392
Total cholesterol (mg/dl)	255.8 ±51.4	267.9 ± 48.9	0.2358
HDL cholesterol (mg/dl)	79.2 ± 26.7	71.4 ± 25.1	0.1391
LDL cholesterol (mg/dl)	153.4 ± 26.1	141.5 ± 27.9	**0.0344**
Tryglycerides (mg/dl)	233.9 ± 75.6	268.8 ± 63.9	**0.0138**
Apoplipoprotein A1 (g/l)	1.89 ± 0.35	1.85 ± 0.47	0.649
Apoplipoprotein B (g/l)	1.31 ± 0.33	1.23 ± 0.27	0.1832
Adiponectin (ng/ml)	4.06 ± 2.33	2.97 ± 1.45	**0.004**
HbA1c (%)	5.16 ± 0.38	5.43 ± 0.49	**0.0042**
Insulin (μ/IU/ml)	5.17 ± 2.9	3.71 ± 1.8	**0.002**
Glucose (mg/dl) 0 h	73.5 ± 7.9	91.3 ± 19.8	**<0.001**
Glucose (mg/dl) 1 h	163.9 ± 25.1	188.7 ± 41.3	**0.0011**
Glucose (mg/dl) 2 h	129.8 ± 34.1	172.5 ± 29.8	**<0.001**
QUICK-IS	0.43 ± 0.27	0.47 ± 0.19	0.3791

BMI, body mass index; GA, gestational age; HDL, high-density lipoprotein; LDL, low-density lipoprotein; QUICK-IS, quantitative insulin sensitivity check index.

### PPARγ expression in the adipose and placenta from healthy pregnant and GDM women

**Figure 1:**
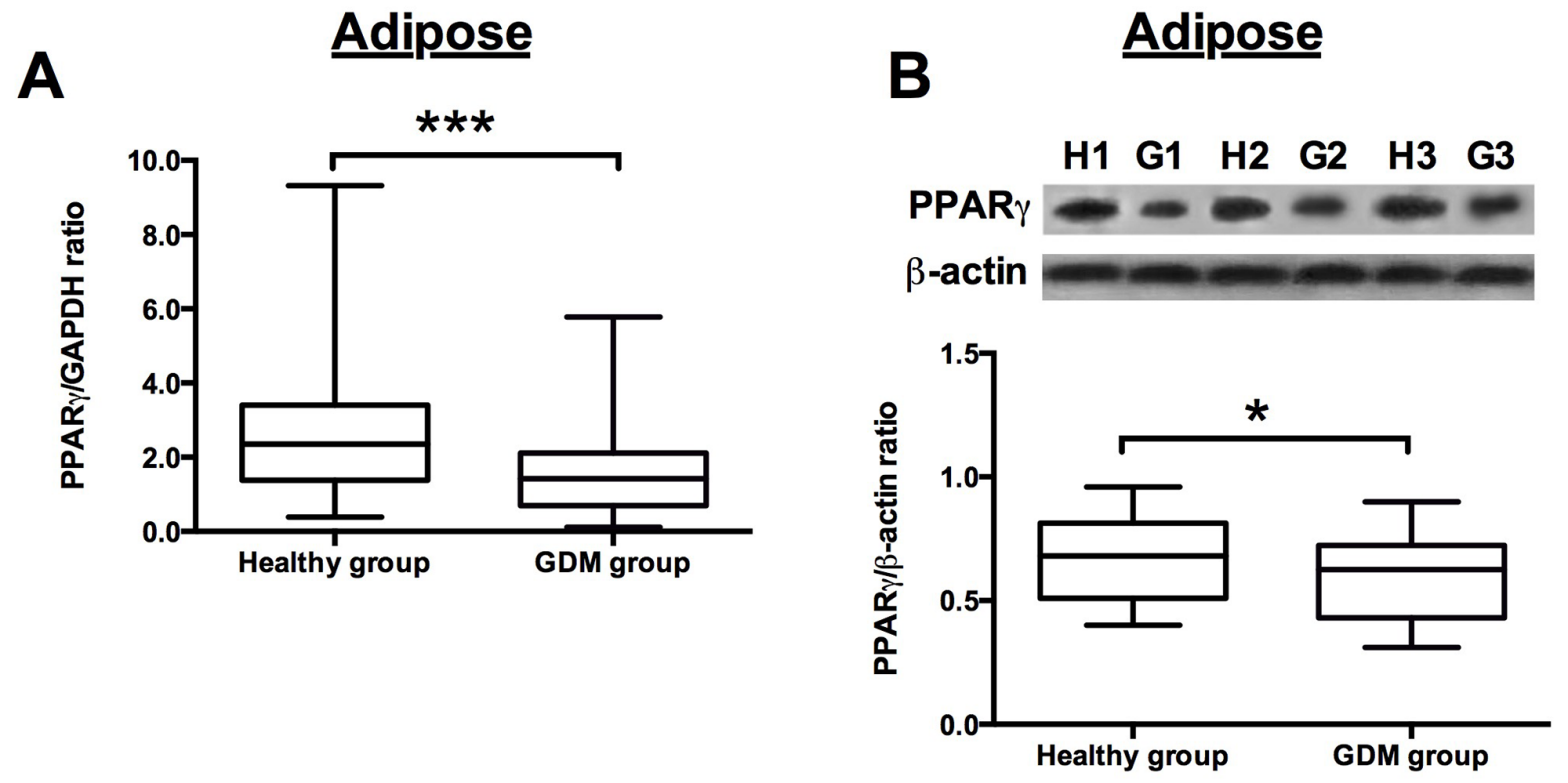
PPARγ expression in the adipose from healthy pregnant and GDM subjects **(A)** The relative expression of PPARγ mRNA in the adipose from healthy pregnant (n = 38) and GDM subjects (n = 66) was determined by qRT-PCR. Differences between groups were analyzed by unpaired *t*-test. Significant differences between groups were indicated as ^***^P<0.001. **(B)** The representative western blotting images (upper panel) of PPARγ in the adipose from 3 healthy pregnant and GDM subjects. H1, H2, H3 represent for healthy pregnant subject 1, 2 and 3, respectively; G1, G2 and G3 represent for GDM subject 1, 2 and 3, respectively; the densitometric analysis (lower panel) of PPARγ protein as measured by western blot in the adipose from healthy pregnant (n =38) and GDM subjects (n = 66). Differences between groups were analyzed by unpaired *t*-test. Significant differences between groups were indicated as ^***^P<0.001.

**Figure 2:**
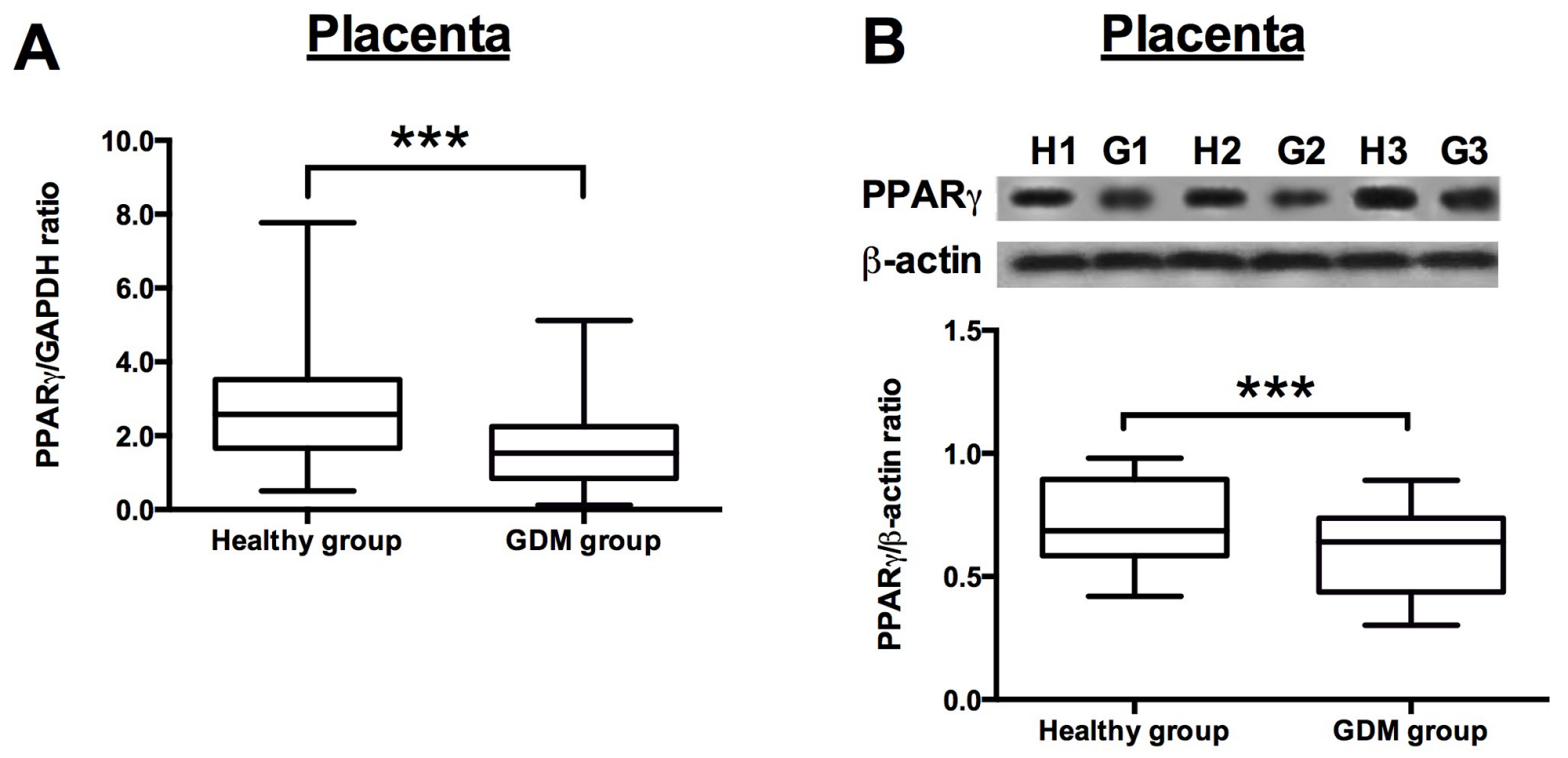
PPARγ expression in the placenta from healthy pregnant and GDM subjects **(A)** The relative expression of PPARγ mRNA in the placenta from healthy pregnant (n = 38) and GDM subjects (n = 66) was determined by qRT-PCR. Differences between groups were analyzed by unpaired *t*-test. Significant differences between groups were indicated as ^***^P<0.001. **(B)** The representative western blotting images (upper panel) of PPARγ in the placenta from 3 healthy pregnant and GDM subjects. H1, H2, H3 represent for healthy pregnant subject 1, 2 and 3, respectively; G1, G2 and G3 represent for GDM subject 1, 2 and 3, respectively; the densitometric analysis (lower panel) of PPARγ protein as measured by western blot in the placenta from healthy pregnant (n = 38) and GDM subjects (n = 66). Differences between groups were analyzed by unpaired *t*-test. Significant differences between groups were indicated as ^***^P<0.001.

The qRT-PCR assay and western blotting assay were performed to examine the PPARγ mRNA and protein expression levels, respectively, in the adipose and placenta from the recruited subjects. The results showed that the PPARγ mRNA expression levels were significantly down-regulated in the GDM women when compared to that in the normal healthy pregnant women (Figure 1A), and western blot assay showed that the protein levels of PPARγ were lower in the adipose tissues from GDM women than from that from healthy pregnant subjects (Figure 1B). In addition, the mRNA and protein expression levels of PPARγ in the placenta tissues were also determined, and consistently, the mRNA and protein expression of PPARγ in the placenta from GDM women were significantly lower than that from healthy pregnant women (Figure 2A and 2B).

### The correlation between adipose PPARγ mRNA expression and the clinical parameters in the GDM women

**Figure 3:**
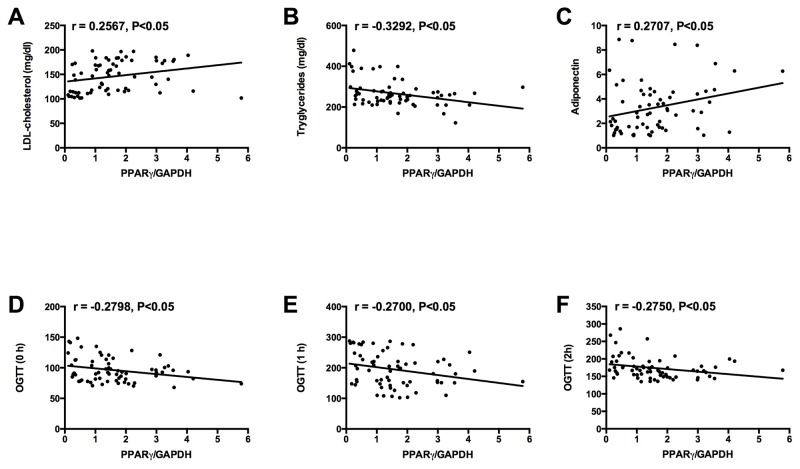
Correlations between adipose PPARγ mRNA expression and **(A)** LDL cholesterol level, **(B)** tryglycerides level, **(C)** adiponectin level, and glucose levels at **(D)** 0 h, **(E)** 1 h, and **(F)** 2 h of 75 g OGTT in GDM group.

Univariate correlation analysis using the Spearman correlation analysis was performed to examine the correlation between adipose PPARγ mRNA expression and the clinical parameters in the GDM women, and the results were shown in Table 2. The mRNA expression level of PPARγ was positively correlated with LDL cholesterol and adiponectin levels in the GDM women (Table 2, Figure 3A and 3C). In addition, the mRNA expression level of PPARγ was negatively correlated with tryglycerides levels and glucose levels at 0 h, 1 h, and 2 h of 75 g OGTT in GDM women (Table 2, Figure 3B, 3D, 3E and 3F). No significant correlation was observed between adipose PPARγ mRNA expression and the other clinical parameters in the GDM women (Table 2).

**Table 2:** Univariate correlations between adipose PPARγ mRNA expression and clinical parameters of GDM subjects

Parameters	PPARγ levels in adipose tissues	
	r value	P value
Age (years)	0.132	0.6798
Pre-pregnancy BMI (kg/m2)	-0.112	0.1981
Pregnancy BMI (kg/m2)	-0.215	0.099
Body weight gain (kg)	0.334	0.3219
GA at delivery (wks)	0.026	0.4589
Fetal weight (g)	-0.136	0.3324
Total cholesterol (mg/dl)	0.412	0.5567
HDL cholesterol (mg/dl)	0.199	0.1562
LDL cholesterol (mg/dl)	0.2567	**0.0375**
Tryglycerides	-0.3292	**0.007**
Apoplipoprotein A1 (g/l)	0.023	0.321
Apoplipoprotein B (g/l)	0.117	0.432
Adiponectin (ng/ml)	0.2707	**0.0279**
HbA1c (%)	-0.039	0.069
Insulin (μ/IU/ml)	0.119	0.075
Glucose (mg/dl) 0 h	-0.2798	**0.0229**
Glucose (mg/dl) 1 h	-0.27	**0.0284**
Glucose (mg/dl) 2 h	-0.275	**0.0255**
QUICK-IS	-0.023	0.453

BMI, body mass index; GA, gestational age; HDL, high-density lipoprotein; LDL, low-density lipoprotein; QUICK-IS, quantitative insulin sensitivity check index.

### The correlation between placenta PPARγ mRNA expression and the clinical parameters in the GDM women

**Figure 4:**
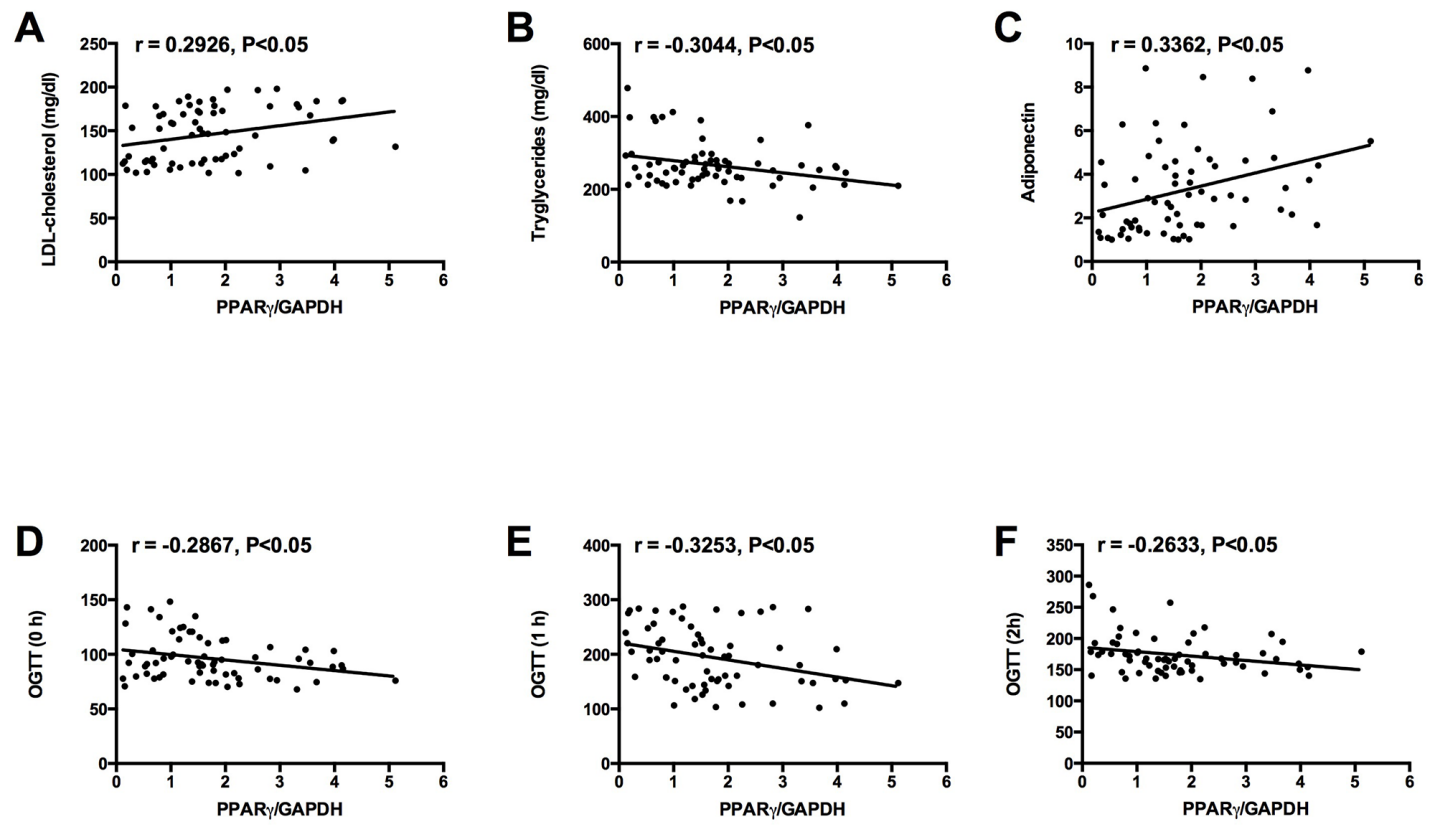
Correlations between placenta PPARγ mRNA expression and **(A)** LDL cholesterol level, **(B)** tryglycerides level, **(C)** adiponectin level, and glucose levels at **(D)** 0 h, **(E)** 1 h, and **(F)** 2 h of 75 g OGTT in GDM group.

Similarly, the correlation between placenta PPARγ mRNA expression and clinical parameters in the GDM women were also investigated and the results were shown in Table 3. The mRNA expression level of PPARγ was positively correlated with LDL cholesterol and adiponectin levels in the GDM women (Table 3, Figure 4A and 4C). In addition, the mRNA expression level of PPARγ was negatively correlated with tryglycerides levels and glucose levels at 0 h, 1 h, and 2 h of 75 g OGTT in GDM women (Table 2 and Figure 4B, 4D, 4E and 4F). No significant correlation was observed between placenta PPARγ mRNA expression and the other clinical parameters in the GDM women (Table 3).

**Table 3:** Univariate correlations between placenta PPARγ mRNA expression and clinical parameters of GDM subjects

Parameters	PPARγ levels in placenta tissues	
	r value	P value
Age (years)	0.167	0.3589
Pre-pregnancy BMI (kg/m2)	-0.229	0.2467
Pregnancy BMI (kg/m2)	-0.339	0.118
Body weight gain (kg)	0.227	0.4455
GA at delivery (wks)	0.039	0.3245
Fetal weight (g)	-0.336	0.1986
Total cholesterol (mg/dl)	0.447	0.5134
HDL cholesterol (mg/dl)	0.286	0.2598
LDL cholesterol (mg/dl)	0.2926	**0.0171**
Tryglycerides	-0.3044	**0.013**
Apoplipoprotein A1 (g/l)	0.178	0.414
Apoplipoprotein B (g/l)	0.217	0.053
Adiponectin (ng/ml)	0.3362	**0.0058**
HbA1c (%)	-0.305	0.119
Insulin (μ/IU/ml)	0.227	0.097
Glucose (mg/dl) 0 h	-0.2867	**0.0196**
Glucose (mg/dl) 1 h	-0.3253	**0.0077**
Glucose (mg/dl) 2 h	-0.2633	**0.0327**
QUICK-IS	-0.083	0.453

BMI, body mass index; GA, gestational age; HDL, high-density lipoprotein; LDL, low-density lipoprotein; QUICK-IS, quantitative insulin sensitivity check index.

## DISCUSSION

Pregnant women with GDM are at an increased risk of developing preeclampsia and delivering macrosomic infant [18]; and also are prone to developing type 2 diabetes mellitus and cardiovascular diseases after pregnancy [19]. For the newborns, they had an increased risk of developing neonatal hypoglycaemia, hypocalcaemia, polycythemia, respiratory distress syndrome [20], and they also are prone to develop obesity and abnormal glucose metabolism [21]. Because of poor availability of metabolic tissues from pregnant women, there is very limited knowledge about the significance of PPARγ in GDM. Studies have demonstrated the role of PPARγ in the normal placental development and trophoblast differentiation and invasion in gestational tissues [22, 23]. However, it would be helpful for us to have a better understanding of the PPARγ underlying the pathophysiology of GDM if more clinical samples can be collected from pregnant women for examination. As PPARγ is involved in glucose and lipid metabolism in type 2 diabetes mellitus [24, 25], it is probable that PPARγ may also play important roles in the GDM. In the present study, decreased expression of PPARγ was observed in both adipose tissues and placenta tissues from patients with GDM. Consistently, previous studies have shown that the expression of PPARγ was down-regulated in the adipose tissues from obese GDM women [16]. These results may suggest that the down-regulation of PPARγ may be an important modulator in the development of GDM.

The decreased expression of PPARγ was also observed in the patients with GDM, and this finding was consistent with previous reports showing down-regulation of placenta PPARγ expression under mild hyperglycaemia in GDM women and streptozotocin-induced diabetic rats [26, 27]. However, in mice study, PPARγ expression was found to be up-regulated in placentas of diabetic pregnant mice with severe hyperglycaemia [28]. In addition, the expression of PPARγ in leukocyte was significantly higher in GDM women than that in healthy pregnant women, and the study suggested that leukocyte PPARγ overexpression may be a regulatory adaption of the maternal organism to increased oxidative stress during diabetic pregnancy [17]. These discrepancies may partly result tissue-specific or species-specific differences in PPARγ expression. Therefore, in the future studies, it is necessary to examine the expression of PPARγ under different glycaemic conditions in different species or cells to resolve the contradictions. In our study, the glucose levels at 0 h, 1 h and 2 h of OGTT in GDM women were significantly higher than that in healthy pregnant women, while fast insulin levels were lower in GDM women. The expression of PPARγ in adipose and placenta from GDM women were negatively correlated with the glucose levels at 0 h, 1 h and 2 h of OGTT. On the contrary, previous studies showed that the expression of PPARγ in leukocyte was positively correlated with glucose levels at 0 h, 1 h and 2 h of OGTT in GDM women, and no correlation between leukocyte PPARγ expression and insulin levels in GDM women [17]. More studies should be performed to further elucidate these discrepancies. In terms of glucose metabolism, the synthetic agonists of PPARγ, thiazolidinediones, have been shown to improve glucose tolerance by enhancing insulin sensitivity and restoring the function of β-cells in diabetic subjects [29], and patients with a dominant-negative mutation in the PPARγ genes showed severe hyperglycemia, suggesting the important role of PPARγ in regulating glucose homeostasis [13]. In this regard, the reduced glucose tolerance of GDM women in the present study may be associated with regulatory role of PPARγ in glucose metabolism, which requires further mechanistic investigations.

The lipid and lipoprotein metabolism has been shown to be affected in the GDM women [30]. In the present study, we found that the levels of LDL cholesterol and adiponectin were significantly lower, and the levels of tryglycerides were significantly higher in GDM women than that in healthy pregnant women. Consistently, studies from Koukkou et al., showed that LDL cholesterol was decreased and tryglycerides was increased in GDM women [31]. Capobianco et la., showed that maternal serum adiponectin concentrations were significantly lower in GDM patients compared with patients with normal glucose tolerance [32]. In addition, we also found that the expression of PPARγ in both adipose and placenta tissues from GDM women was negatively correlated with trygylcerides levels, and positively correlated with LDL cholesterol and adiponectin levels, suggesting PPARγ may be involved the altered metabolism of lipid and lipoprotein in GDM women. However, the relationship between PPARγ expression and altered metabolism of lipid and lipoprotein in GDM women may require further examination. Both *in vitro* and *in vivo* studies have demonstrated that PPARγ played important roles in the transcriptional cascade underlying adipocyte differentiation [33, 34], and PPARγ also was essential for the entraining of adipose tissue lipid metabolism to nutritional state [35]. More importantly, PPARγ was found to promote futile cycling in adipocytes between triglyceride esterification and de-esterification [36]. Taken together, the significant correlations between PPARγ and LDL cholesterol, adiponectin and tryglycerides may be due to role of PPARγ in the cellular assimilation of lipids via anabolic pathways.

In conclusion, we demonstrated the down-regulation of PPARγ in both adipose and placenta tissues from GDM women, and we showed for the first time that expression of PPARγ in both adipose and placenta tissues was negatively correlated with hyperglycaemia. Our studies also suggested that PPARγ may be involved in the altered glucose metabolism, lipid and lipoprotein metabolism in the GDM women. Further studies are required to fully understand the role of PPARγ underlying the pathophysiology of GDM.

## MATERIALS AND METHODS

### Subject recruitment

In the present study, a total of 104 pregnant women between 26-37 weeks of gestation were recruited at the Shenzhen People’s Hospital, Shenzhen, China. The age range between 22-39 years old. All the clinical investigations were approved by the Bioethics Committee of the Shenzhen People’s Hospital and were conducted in accordance with the guidelines in the Declaration of Helsinki. Informed consent was obtained from all the recruited subjects. The GDM was diagnosed if one or more plasma glucose levels were elevated during a 75 g, 2 h oral glucose tolerance test (OGTT) according to the criteria set by WHO [37]. Among all the recruited subjects, 66 subjects were diagnosed with GDM, and 38 subjects were healthy pregnant women. The inclusion criteria for this study were the following: no GDM in the previous pregnancy; no family history of diabetes in the first-degree relatives; not taking insulin or oral hypoglycaemic medications; absence of any form of the pre-pregnancy diabetes; no control by diet and exercise before the overnight fast.

### Adipose and placenta tissues collection

The subcutaneous adipose tissue and term placental tissues were obtained from all recruited subjects after Cesarean section at term under a continuous lumbar epidural infusion of local anesthetic in the Department of Obstetrics at the Shenzhen People’s Hospital. The placental villous explants were obtained after the basal and the chorial plates were dissected out from central cotyledons. The adipose tissues and placental villous explants were immediately snap-frozen in liquid nitrogen and stored in -80 ^o^C for further analysis.

### Quantitative real-time PCR (qRT-CPR)

Total RNA from adipose tissues or placenta tissues was extracted by using the TRIzol reagent (Invitrogen, Carlsbad, USA) according to the manufacturer’s instructions. Total RNA was reverse transcribed into cDNA by using the Reverse Transcription System Kit (Applied Biosystems, Illinois, USA). The real time PCR was performed with an Applied Biosystems Prims7500 Fast Sequence Detection Sysem using TaqMan universal PCR master mix according to the manufacturer’s instructions (Applied Biosystems). The mRNA expression levels of PPARγ were normalized to GAPDH. The primers of PPARγ were as follow: forward: 5’-GGGATCAGCTCCGTGGATCT-3’; reverse: 5’-TGCACTTTGGTACTCTTGAAGTT-3’. The primers of GAPDH were as follow: forward: 5’-GCACCGTCAAGGCTGAGAAC-3’; reverse: 5’-TGG TGAAGACGCCAGTGGA-3’. The relative expression levels of PPARγ was calculated based on the 2^-Δ;ΔCt^ method.

### Western blot

The proteins from the tissue samples were extracted by using the ice-cold lysis buffer with protease inhibitor cocktails. The extracted proteins were separated with the use of SDS-polyacrylamide gel electrophoresis. Proteins were then transferred to a nitrocellulose membrane and the membrane was incubated with 1% BSA in PBST at room temperature for 1 h. Then the membrane was further incubated with polyclonal rabbit anti-PPARγ antibodies (1:1500; Abcam, Cambridge, USA) and monoclonal rabbit anti-β-actin (1:3000; used as internal control, Abcam) at 4 °C overnight. The membrane was washed and further incubated with HPR-conjugated secondary antibodies. The bands of proteins were detected by using the Western Blotting Luminal Reagent (Thermo Fisher Scientific) according to manufacturer’s instructions.

### Anthropometric and biochemical measurements

The recruited subjects gave information on their maternal age and pre-pregnancy weight. The weight and height of patients during the third trimester of pregnancy and the fetal weight were measured by standard methods, and both body again and pre-pregnancy body mass index (BMI) expressed as weight before pregnancy divided by height square were calculated.

Blood samples were drawn after a 12 h overnight fast. Serum total cholesterol, HDL-cholesterol, LDL cholesterol and triglycerides were determined by the total cholesterol CHOD-PAP and triglyceride GPO-PAP kits (Roche, Mannheim, Germany). Apoplipoprotein A1, apoplipoprotein B, and adiponetcin concentrations were measured by enzyme-linked immunosorbent assay method (AssayPro, St. Charles, USA). The glycated haemoglobin (HbA_1c_) was measured by a latex enhanced turbidimetric immunoassay using specific monoclonal antibodies. Plasma insulin was quantified using Elecsys insulin assay (Roche). To assess insulin sensitivity, the quantitative insulin check index (QUICKI-IS) was calculated as follow: QUIKI = 1/[log(I0) + log (G0)], where I0 is the fasting plasma insulin (μU/ml) and G0 is the fasting blood glucose concentration (mg/dl) [38].

### Statistical analysis

All the statistical analysis and graphs plotting were performed by using GraphPad Prism Version 6.0 software. All the data were presented as mean ± standard deviation. Differences between the two groups, including clinical parameters and expression data were analyzed by unpaired Student’s *t*-test. Relationship between PPARγ mRNA expression and clinical parameters were determined by the nonparametric test of Spearman’s rank correlation coefficient. P values less than 0.05 were considered to be statistically significant.

## References

[1] Chiefari E, Arcidiacono B, Foti D, Brunetti A (2017). Gestational diabetes mellitus: an updated overview. J Endocrinol Invest.

[2] Mericq V, Martinez-Aguayo A, Uauy R, Iniguez G, Van der Steen M, Hokken-Koelega A (2017). Long-term metabolic risk among children born premature or small for gestational age. Nat Rev Endocrinol.

[3] Barbour LA, McCurdy CE, Hernandez TL, Kirwan JP, Catalano PM, Friedman JE (2007). Cellular mechanisms for insulin resistance in normal pregnancy and gestational diabetes. Diabetes care.

[4] Colomiere M, Permezel M, Lappas M (2010). Diabetes and obesity during pregnancy alter insulin signalling and glucose transporter expression in maternal skeletal muscle and subcutaneous adipose tissue. J Mol Endocrinol.

[5] Retnakaran R, Hanley AJ, Raif N, Connelly PW, Sermer M, Zinman B (2004). Reduced adiponectin concentration in women with gestational diabetes: a potential factor in progression to type 2 diabetes. Diabetes Care.

[6] Ranheim T, Haugen F, Staff AC, Braekke K, Harsem NK, Drevon CA (2004). Adiponectin is reduced in gestational diabetes mellitus in normal weight women. Acta Obstet Gynecol Scand.

[7] Lv X, Zhang L, Sun J, Cai Z, Gu Q, Zhang R, Shan A (2017). Interaction between peroxisome proliferator-activated receptor gamma polymorphism and obesity on type 2 diabetes in a Chinese Han population. Diabetol Metab Syndr.

[8] Majid M, Masood A, Kadla SA, Hameed I, Ganai BA (2017). Association of Pro12Ala polymorphism of peroxisome proliferator-activated receptor gamma 2 (PPARgamma2) gene with type 2 diabetes mellitus in ethnic kashmiri population. Biochem Genet.

[9] Raj R, Bhatti JS, Bhadada SK, Ramteke PW (2017). Association of polymorphisms of peroxisome proliferator activated receptors in early and late onset of type 2 diabetes mellitus. Diabetes Metab Syndr.

[10] He W, Barak Y, Hevener A, Olson P, Liao D, Le J, Nelson M, Ong E, Olefsky JM, Evans RM (2003). Adipose-specific peroxisome proliferator-activated receptor gamma knockout causes insulin resistance in fat and liver but not in muscle. Proc Natl Acad Sci U S A.

[11] Camacho A, Huang JK, Delint-Ramirez I, Yew Tan C, Fuller M, Lelliott CJ, Vidal-Puig A, Franklin RJ (2013). Peroxisome proliferator-activated receptor gamma-coactivator-1 alpha coordinates sphingolipid metabolism, lipid raft composition and myelin protein synthesis. Eur J Neurosci.

[12] Olefsky JM (2000). Treatment of insulin resistance with peroxisome proliferator-activated receptor gamma agonists. J Clin Invest.

[13] Barroso I, Gurnell M, Crowley VE, Agostini M, Schwabe JW, Soos MA, Maslen GL, Williams TD, Lewis H, Schafer AJ, Chatterjee VK, O’Rahilly S (1999). Dominant negative mutations in human PPARgamma associated with severe insulin resistance, diabetes mellitus and hypertension. Nature.

[14] Jiang C, Ting AT, Seed B (1998). PPAR-gamma agonists inhibit production of monocyte inflammatory cytokines. Nature.

[15] Capobianco E, Martinez N, Fornes D, Higa R, Di Marco I, Basualdo MN, Faingold MC, Jawerbaum A (2013). PPAR activation as a regulator of lipid metabolism, nitric oxide production and lipid peroxidation in the placenta from type 2 diabetic patients. Mol Cell Endocrinol.

[16] Catalano PM, Nizielski SE, Shao J, Preston L, Qiao L, Friedman JE (2002). Downregulated IRS-1 and PPARgamma in obese women with gestational diabetes: relationship to FFA during pregnancy. Am J Physiol Endocrinol Metab.

[17] Wojcik M, Mac-Marcjanek K, Nadel I, Wozniak L, Cypryk K (2015). Gestational diabetes mellitus is associated with increased leukocyte peroxisome proliferator-activated receptor gamma expression. Arch Med Sci.

[18] Hay WW (2012). Care of the infant of the diabetic mother. Curr Diab Rep.

[19] Burlina S, Dalfra MG, Chilelli NC, Lapolla A (2016). Gestational diabetes mellitus and future cardiovascular risk: an Update. Int J Endocrinol.

[20] Flores-le Roux JA, Sagarra E, Benaiges D, Hernandez-Rivas E, Chillaron JJ, Puig de Dou J, Mur A, Lopez-Vilchez MA, Pedro-Botet J (2012). A prospective evaluation of neonatal hypoglycaemia in infants of women with gestational diabetes mellitus. Diab Res Clin Pract.

[21] Logan KM, Gale C, Hyde MJ, Santhakumaran S, Modi N (2017). Diabetes in pregnancy and infant adiposity: systematic review and meta-analysis. Arch Dis Child Fetal Neonatal Ed.

[22] Barak Y, Nelson MC, Ong ES, Jones YZ, Ruiz-Lozano P, Chien KR, Koder A, Evans RM (1999). PPAR gamma is required for placental, cardiac, and adipose tissue development. Mol Cell.

[23] Tarrade A, Schoonjans K, Pavan L, Auwerx J, Rochette-Egly C, Evain-Brion D, Fournier T (2001). PPARgamma/RXRalpha heterodimers control human trophoblast invasion. J Clin Endocrinol Metab.

[24] Lu P, Zhao Z (2017). Advances on PPARgamma research in the emerging era of precision medicine. Curr Drug Targets.

[25] Ishibashi K, Takeda Y, Nakatani E, Sugawara K, Imai R, Sekiguchi M, Takahama R, Ohkura N, Atsumi GI (2017). Activation of PPARgamma at an early stage of differentiation enhances adipocyte differentiation of MEFs derived from type II diabetic TSOD mice and alters lipid droplet morphology. Biol Pharm Bull.

[26] Capobianco E, Jawerbaum A, Romanini MC, White V, Pustovrh C, Higa R, Martinez N, Mugnaini MT, Sonez C, Gonzalez E (2005). 15-Deoxy-Delta12,14-prostaglandin J2 and peroxisome proliferator-activated receptor gamma (PPARgamma) levels in term placental tissues from control and diabetic rats: modulatory effects of a PPARgamma agonist on nitridergic and lipid placental metabolism. Reprod Fertil Dev.

[27] Jawerbaum A, Capobianco E, Pustovrh C, White V, Baier M, Salzberg S, Pesaresi M, Gonzalez E (2004). Influence of peroxisome proliferator-activated receptor gamma activation by its endogenous ligand 15-deoxy Delta12,14 prostaglandin J2 on nitric oxide production in term placental tissues from diabetic women. Mol Hum Reprod.

[28] Suwaki N, Masuyama H, Masumoto A, Takamoto N, Hiramatsu Y (2007). Expression and potential role of peroxisome proliferator-activated receptor gamma in the placenta of diabetic pregnancy. Placenta.

[29] Cavaghan MK, Ehrmann DA, Byrne MM, Polonsky KS (1997). Treatment with the oral antidiabetic agent troglitazone improves beta cell responses to glucose in subjects with impaired glucose tolerance. J Clin Invest.

[30] Herrera E, Desoye G (2016). Maternal and fetal lipid metabolism under normal and gestational diabetic conditions. Horm Mol Biol Clin Investigation.

[31] Koukkou E, Watts GF, Lowy C (1996). Serum lipid, lipoprotein and apolipoprotein changes in gestational diabetes mellitus: a cross-sectional and prospective study. J Clin Pathol.

[32] Pala HG, Ozalp Y, Yener AS, Gerceklioglu G, Uysal S, Onvural A (2015). Adiponectin levels in gestational diabetes mellitus and in pregnant women without glucose intolerance. Adv Clin Exp Med.

[33] Sandouk T, Reda D, Hofmann C (1993). Antidiabetic agent pioglitazone enhances adipocyte differentiation of 3T3-F442A cells. Am J Physiol.

[34] Rosen ED, Sarraf P, Troy AE, Bradwin G, Moore K, Milstone DS, Spiegelman BM, Mortensen RM (1999). PPAR gamma is required for the differentiation of adipose tissue *in vivo* and *in vitro*. Mol Cell.

[35] Semple RK, Chatterjee VK, O’Rahilly S (2006). PPAR gamma and human metabolic disease. J Clin Invest.

[36] Guan HP, Li Y, Jensen MV, Newgard CB, Steppan CM, Lazar MA (2002). A futile metabolic cycle activated in adipocytes by antidiabetic agents. Nature Med.

[37] Bertero T, Grosso S, Robbe-Sermesant K, Lebrigand K, Henaoui IS, Puissegur MP, Fourre S, Zaragosi LE, Mazure NM, Ponzio G, Cardinaud B, Barbry P, Rezzonico R (2012). “Seed-Milarity” confers to hsa-miR-210 and hsa-miR-147b similar functional activity. PLoS One.

[38] Katz A, Nambi SS, Mather K, Baron AD, Follmann DA, Sullivan G, Quon MJ (2000). Quantitative insulin sensitivity check index: a simple, accurate method for assessing insulin sensitivity in humans. J Clin Endocrinol Metabol.

